# Undergraduate nursing students' clinical adaptation: a qualitative journey mapping study from student and clinical preceptor perspectives

**DOI:** 10.3389/fmed.2026.1966449

**Published:** 2026-10-09

**Authors:** Chuanqin Wu, Qingqing Hong, Yang Hao, Haiyu Yao, Yuanyuan Liang, Jiaqian Chen, Zhijun Shu, Man Wu, Shaohua Hu

**Affiliations:** 1 School of Nursing, Anhui Medical University, Hefei, China; 2 Department of Nursing, The First Affiliated Hospital of Anhui Medical University, Hefei, China; 3 Department of Medical Oncology, The First Affiliated Hospital of Anhui Medical University, Hefei, China

**Keywords:** clinical adaptation, clinical education, clinical preceptorship, journey mapping, qualitative research, undergraduate nursing students

## Abstract

**Background:**

Clinical adaptation is shaped by students' readiness, precepting arrangements, patient responses, and ward conditions. Previous studies have usually examined student and preceptor experiences separately. This study aimed to explore undergraduate nursing students' adaptation to clinical practice from student and clinical preceptor perspectives and to identify key touchpoints, differences in focus, and support needs through journey mapping.

**Methods:**

A descriptive qualitative study was conducted. Sixteen undergraduate nursing students and 11 clinical preceptors from a tertiary teaching hospital in Anhui Province, China, were purposively recruited. Individual face-to-face semi-structured interviews were analysed using Braun and Clarke's thematic analysis. Student and preceptor data were analysed separately and then compared at the thematic level. Journey mapping was then used to organise the findings across the clinical adaptation process and to present students' experiences and clinical preceptors' observations within the same journey map.

**Results:**

Three themes and seven subthemes were identified: Positioning and Participation, Interaction and Adjustment, and Readjustment and Identity. Students judged their adaptation mainly by whether they could take part in patient care and whether their involvement was accepted by preceptors and patients. Preceptors adjusted participation and supervision according to observed performance, task risk, patient preferences, and safety requirements. Feedback, procedural setbacks, and ward rotations could interrupt participation, and previously established progress and support did not always carry over.

**Conclusions:**

Clinical adaptation was described as involving repeated adjustments as opportunities to participate were gained, interrupted, and re-established. Clinical education should support continuity of participation, provide specific feedback and opportunities to re-engage after setbacks, and improve the transfer of learning information across ward rotations while maintaining patient safety.

## 1 Introduction

Clinical practice is an important stage in which undergraduate nursing students apply classroom knowledge to the care of real patients and gradually develop an understanding of their clinical roles and responsibilities (1, 2). During clinical practice, students need to become familiar with ward environments and workflows under the guidance of clinical preceptors and take part in nursing procedures, communication with patients, and observation of patients’ conditions (3, 4). They also need to understand their role as learners, the scope of their participation, and their responsibilities in patient care (5). Appropriate opportunities to participate, together with guidance and feedback, can help students gain experience in patient care and build clinical confidence. When these conditions are insufficient, students may feel that they are not yet prepared to meet clinical demands (6, 7). Nursing students' clinical adaptation involves not only developing clinical knowledge and skills, but also understanding and adjusting to their learner role, scope of participation, and responsibilities in patient care. How nursing students adapt to these demands and the support available to them is therefore an important concern in clinical nursing education.

Unlike predefined teaching cases used in the classroom, clinical learning involves patients with different care needs, actual workflows, and changing clinical situations, and is often embedded in everyday patient care (1, 3). Nursing students generally expect to gain experience through observation, questioning, and practice. However, the learning opportunities available to them and the extent to which they can participate are not always clearly defined or consistent (8, 9). When the purpose of a task and its learning requirements are not explained, students may find it difficult to understand how the task relates to their clinical learning (10). Limited practical experience, concerns about making mistakes, and fear of criticism may also make students less willing to take the initiative or raise questions (11, 12). For tasks involving patient safety or greater procedural risk, preceptors usually consider students' experience and readiness when assigning them to observe, assist, or perform the task under supervision (8, 13). Students' gradual participation in clinical tasks depends not only on their own experience and readiness, but also on preceptors' judgements, patient safety requirements, and working conditions within the ward (14).

The pressures nursing students face during clinical practice do not remain constant but change with the stage of their placement and the extent of their participation (15). At the beginning of clinical practice, unfamiliar ward environments, contact with real patients, and concerns about making mistakes often cause varying levels of anxiety (16). Students with higher levels of anxiety may focus more on avoiding errors and confirming whether they are permitted to participate, making them more cautious when observing, asking questions, and practising procedures (17). As clinical practice progresses, their unfamiliarity with the environment may lessen, but the pressure they experience does not necessarily continue to decline. Previous research has found that students' concerns may shift from early unease about encountering patients and working in unfamiliar environments to worries about insufficient professional knowledge and clinical skills (15). With increasing clinical experience, students begin to look beyond individual procedures and develop a broader understanding of patient safety, teamwork, communication with patients, and the clinical work environment (18, 19). During this process, timely and specific explanations and feedback can help students understand clinical expectations, recognise their own progress, and develop a clearer understanding of the nursing role and their readiness for future practice (20–23).

Changes in nursing students' emotions, competence, and understanding of their roles are often reflected in specific clinical teaching interactions (24, 25). During clinical practice, preceptors not only demonstrate nursing procedures and provide practical guidance but also adjust how students participate according to their performance, the risks associated with the task, and patient safety considerations (13). Preceptors can also observe how students are adapting through their performance in nursing procedures, communication and interactions, and responses to feedback (24). However, clinical preceptorship usually takes place alongside demanding patient care. Heavy workloads, limited time for teaching, and insufficient organisational support may affect preceptors' ability to provide ongoing guidance and feedback (26, 27). Existing studies have mainly focused on nursing students' stress, role transition, and professional identity, or on preceptors' experiences of providing clinical guidance (26, 28–30). Few studies have followed the course of clinical practice while bringing together students' own experiences and preceptors' observations. Including the perspectives of both nursing students and preceptors may therefore provide a fuller understanding of how students adapt during clinical practice.

Nursing students' adaptation changes as clinical practice progresses (15, 24). A method is therefore needed to integrate students' experiences and preceptors' observations across the course of clinical practice. Journey mapping follows the course of an experience and can be used to organise key touchpoints, interactions, emotional changes, difficulties, and needs across service, care, or learning processes (31). This method has mainly been used to examine patient experiences and improve health services, while its application in nursing education remains limited; existing studies have also focused largely on single learner groups (31, 32). Placing students' experiences and preceptors' observations on the same clinical practice timeline can show key experiences and support needs at different stages and allow comparison of how the two groups understand the process (33). Accordingly, this study aimed to develop a journey map of clinical adaptation from the perspectives of nursing students and clinical preceptors, identify key experiences, major difficulties, and support needs across different stages of clinical practice, and compare areas of shared concern and differences in how the two groups understood this process.

## 2 Materials and methods

### 2.1 Study design

This study used a descriptive qualitative design. Data were collected through semi-structured interviews conducted after the clinical placement had ended. Undergraduate nursing students were asked to reflect on their experiences during the placement, while clinical preceptors were asked to describe their observations of students during the placement. Student and preceptor data were analysed separately using thematic analysis and then compared at the thematic level to identify shared concerns and differences in understanding. After the thematic framework was developed, journey mapping was used to organise the findings across the clinical adaptation process and to present students' experiences and clinical preceptors' observations within the same journey map. The reporting of this study followed the Consolidated Criteria for Reporting Qualitative Research (COREQ) checklist (34), with the completed checklist provided in Supplementary File S1.

### 2.2 Setting and participants

The study was conducted at a tertiary Grade A general teaching hospital in Anhui Province, China. The student participants were all final-year undergraduate nursing students from the same cohort. They completed eight months of clinical placement from October 2025 to May 2026, following the hospital's ward rotation schedule. Each student rotated through eight clinical units, although the specific units varied among students. Across participants, the rotations included medical, surgical, emergency, intensive care, operating room, oncology, paediatric, and obstetric and gynaecological units. The preceptor participants were registered nurses at the same hospital who had at least three years of experience precepting undergraduate nursing students.

### 2.3 Sampling and recruitment

Purposive sampling was used. Recruitment information for nursing students was posted in the student internship WeChat group by a staff member of the hospital nursing department. Recruitment information for clinical preceptors was forwarded by the nursing department to relevant clinical units, which assisted in circulating it. The recruitment information provided the contact details of a member of the research team, and interested individuals contacted the research team directly. The researchers then assessed their eligibility and explained the study and interview arrangements through WeChat or by telephone. The nursing department, head nurses, and clinical preceptors were not involved in participant selection and were not informed of the final participant list. Nursing students and clinical preceptors were recruited separately and were not matched one-to-one. No eligible participants who contacted the research team declined participation or withdrew from the study.

Recruitment, data collection, and preliminary analysis proceeded concurrently. Data saturation was assessed separately for the student and clinical preceptor interviews. The research team reviewed the preliminary analysis throughout data collection and jointly assessed whether saturation had been reached. Saturation was judged based on changes at both the code and theme levels. It was provisionally considered to have been reached when additional interviews generated no new codes relevant to the research question and made no substantial additions to the existing themes. Saturation was provisionally reached after the 14th student interview and the 9th clinical preceptor interview. Two additional participants were then interviewed in each group to further confirm saturation. These additional interviews did not generate any new themes, and recruitment was therefore stopped. The final sample comprised 16 undergraduate nursing students and 11 clinical preceptors.

### 2.4 Interviews

Data were collected through semi-structured interviews. The interview guides were developed from the study aim and relevant literature (1, 35, 36). They were also informed by the stages of clinical practice, key events, interactions, emotional changes, and support needs commonly examined in journey mapping (37). Separate interview guides were prepared for nursing students and clinical preceptors. The student interviews explored experiences of entering clinical practice, participation in nursing work and patient care, interactions with preceptors, patients, and family members, significant events during practice, changes over time, and support needs. The preceptor interviews explored their observations of students' experiences and changes during clinical practice, their decisions regarding student participation and supervision, and the challenges they encountered in clinical teaching. The preliminary interview guides were discussed within the research team and reviewed by three experts in nursing education and clinical preceptorship. Before formal data collection, pilot interviews were conducted with two nursing students and two clinical preceptors. Based on their feedback, the wording of several questions and the approach to follow-up probing were revised. The pilot interview data were not included in the final analysis. The main interview questions are presented in Table 1.

**Table 1:** Main questions in the semi-structured interview guides.

Questions for Nursing Students	Questions for Clinical Preceptors
1. Thinking back to when you first started clinical practice and entered a ward, what were you hoping for or worried about? What stood out to you most when you first arrived?	1. What do you usually notice when nursing students first enter the ward, and what difficulties do they tend to face? How do you help them begin to adjust?
2. During clinical practice, how did you move from observing to taking part in nursing work and patient care? What had the greatest influence on this process?	2. How do you usually support students in moving from observation to more active participation in nursing work? What influences the way you arrange their participation?
3. Can you describe an experience with a preceptor, patient, or family member that particularly stayed with you? What happened?	3. What do you usually observe when students ask questions, receive feedback, or interact with patients and family members? Is there an experience that particularly stands out?
4. Was there an event that made you feel you were beginning to adapt, or one that was especially discouraging? What happened afterwards?	4. How do you usually respond when a student encounters difficulty in a procedure or communication, has an unsuccessful attempt, or becomes visibly discouraged?
5. When you moved to a new ward, what did you usually have to adjust to again? How did your experiences differ across wards?	5. From the time students enter your ward until they leave, what changes do you usually observe? What tells you that they are beginning to adapt?
6. Looking back on your clinical practice as a whole, how did your feelings, the way you participated, and your understanding of nursing change? What additional support would have been helpful?	6. What kinds of support do you consider most important for students’ clinical adaptation? What difficulties or constraints do you encounter when providing this support?

### 2.5 Data collection

Formal interviews were conducted from July 31 to August 9, 2026, after the students had completed their eight-month clinical placement. The interviews were one-to-one, face-to-face, and semi-structured. No repeat interviews were conducted. All formal interviews were conducted by Chuanqin Wu, a female master's student in nursing. She had received training in qualitative research methods and had previous interviewing experience. Before the interviews, she had no prior relationship with the participating undergraduate nursing students or clinical preceptors. She did not teach or precept the participating undergraduate nursing students and was not involved in assessing their clinical practice or evaluating the clinical preceptors' work performance. Participants were informed that she was a master's student in nursing and were told the purpose of the study before the interview. Interview times were arranged according to participants’ preferences. All interviews took place in a quiet, private room at the study hospital. No other persons were present during the interviews. The interviewer used the interview guide to ask open-ended questions and followed up on participants' responses. Participants were encouraged to describe when events occurred, the clinical context, their responses at the time, any subsequent effects, and the support they received. Each interview lasted 20–40 min and was audio-recorded in full with the participant's consent. After each interview, the interviewer prepared an interview memo to record contextual details and reflect on the interview process and her own responses during the interview.

### 2.6 Data analysis

Interview data were analysed using Braun and Clarke's approach to thematic analysis (38). Data collection, transcription, and preliminary analysis proceeded concurrently. All interviews were conducted in Chinese. Audio recordings were transcribed verbatim within 48 h of each interview and checked against the original recordings by the interviewer. Transcripts were not returned to participants for comment or correction. Coding and analysis were based on the Chinese transcripts. Quotations selected for the manuscript were translated into English by one member of the research team and checked against the original Chinese transcripts by another. Any differences in wording were discussed and revised to preserve the meaning and tone of the participants' accounts. Names, specific clinical units, and other potentially identifying information were removed during transcription and quotation. Interview excerpts quoted in the study were identified only by participant codes, with S01–S16 used for undergraduate nursing students and T01–T11 for clinical preceptors. The de-identified transcripts were imported into NVivo 12 Pro (QSR International) to support data organisation, coding, and retrieval. The analysis followed six phases: familiarisation with the data, generation of initial codes, development of preliminary themes, review of themes, definition and naming of themes, and production of the report.

Two researchers independently read the transcripts and interview memos several times and conducted line-by-line coding of material related to students' clinical adaptation and preceptors' observations. The interview memos included the interviewer's initial reflections on the interview process. The analysis was primarily inductive and focused on the experiences and views explicitly expressed by participants. Coding was based on the interview data without using a predefined theoretical framework. After independent coding, the two researchers compared their initial codes, merged codes with similar meanings, and discussed differences in coding to develop a shared coding framework. No inter-coder agreement coefficient was calculated. Attention was given to key events, participation in clinical tasks, clinical interactions, difficulties in adaptation, student-reported emotional changes, and support needs. Data from undergraduate nursing students and clinical preceptors were coded and synthesised separately during the initial analysis to retain the perspective of each group. Student data reflected their own experiences during clinical placement, whereas clinical preceptor data reflected their observations and judgements about student performance, patient safety, and the preceptorship process. Clinical preceptor accounts were not used to verify whether students' accounts of their experiences were factually accurate. Within each dataset, codes with related meanings were grouped into candidate themes. The two sets of themes were then compared to identify shared concerns and differences in understanding. Any remaining differences in coding or theme development were discussed within the research team, and the original interview data were revisited when needed. The research team then reviewed the themes against the original data, merging, separating, or revising them where needed before agreeing on the final thematic framework. Participants were not asked to review or provide feedback on the final themes. During theme development and review, the research team also examined data that did not fit the preliminary themes or interpretations. The research team kept records of the initial codes, coding changes, and theme revisions to track changes during the analysis.

Once the thematic framework had been developed, the research team constructed the clinical adaptation journey map based on the thematic analysis findings. The team reviewed the original interview data, with particular attention to participants' descriptions of the sequence of their experiences. These were organised as the main touchpoints according to their position in the adaptation process. The main stages of clinical adaptation were then identified based on recurring sequences in participants' accounts and the order of the different touchpoints. These stages represent recurring patterns across the dataset rather than a fixed sequence experienced by every participant. Students' experiences and clinical preceptors' observations were mapped separately to the relevant touchpoints and stages and presented side by side within the same journey map. The journey map also presented key experiences, student-reported responses, major difficulties, and support needs at each stage. Differences in focus between the two groups were compared at the same or similar touchpoints. During the development and revision of the journey map, the research team repeatedly checked the stages, touchpoints, and related content against the original interview data and revised the map accordingly.

### 2.7 Reflexivity and positionality

Chuanqin Wu and Qingqing Hong were both involved in the independent coding of the interview data and theme development. Both are postgraduate nursing students and have received training in qualitative research methods. Chuanqin Wu conducted all formal interviews and was therefore familiar with the interview process and the contexts described by participants, whereas Qingqing Hong was not involved in the interviews and analysed the data independently based on the interview transcripts. Neither researcher was involved in assessing the participating students' clinical practice or evaluating the work performance of the clinical preceptors. Shaohua Hu participated in the subsequent discussion and review of the themes. During theme development, the research team repeatedly checked their interpretations against the original interview data and revised them when needed to reduce the influence of their own experiences on the findings.

### 2.8 Ethical considerations

The study was approved by the Ethics Committee of Anhui Medical University on July 29, 2026 (approval number: KY2026-09-20). Before the interviews, the researchers explained the study purpose, interview arrangements, and procedures for maintaining data confidentiality. Written informed consent and consent for audio recording were obtained from all participants. Participation was voluntary. Declining to participate or withdrawing from the study would not affect students’ clinical practice assessments, preceptorship relationships, or other participant rights. All interview data were de-identified. Audio recordings and transcripts were stored in encrypted files accessible only to the research team.

## 3 Results

A total of 27 participants were included in the study, comprising 16 undergraduate nursing students and 11 clinical preceptors. Participant characteristics are presented in Table 2. Interviews lasted 20–40 min. The thematic analysis identified three themes and seven subthemes, as shown in Table 3.

**Table 2:** Characteristics of the study participants.

Characteristics	Undergraduate nursing students (*n* = 16)	Clinical preceptors (*n* = 11)
Age, years, median (range)	22 (21–23)	35 (29–41)
Gender, *n* (%)		
Female	14 (87.5)	10 (90.9)
Male	2 (12.5)	1 (9.1)
Clinical placement duration, months	8	—
Number of clinical rotations	8	—
Clinical experience, years, median (range)	—	13 (7–19)
Experience as a clinical preceptor, years, median (range)	—	7 (3–11)
Education, *n* (%)		
Bachelor's degree	—	9 (81.8)
Master's degree	—	2 (18.2)
Professional title^a^, *n* (%)		
Junior	—	2 (18.2)
Intermediate	—	7 (63.6)
Associate senior	—	2 (18.2)

a Professional title refers to the professional title level within the Chinese nursing system.

**Table 3:** Themes and subthemes.

Themes	Subthemes
Positioning and Participation	Role Boundaries
	Learning to Participate
Interaction and Adjustment	Feedback Experiences
	Responses from Patients
	Setback and Recovery
Readjustment and Identity	Support Discontinuity
	Professional Identity

Based on the thematic findings and recurring sequences of experiences described in the interview data, the research team constructed a clinical adaptation journey map to present undergraduate nursing students' experiences, clinical preceptors' observations, the main touchpoints, student-reported responses, major difficulties, and support needs (Figure 1). The findings showed that clinical adaptation was not experienced as a steady process during clinical placement. Students became more involved in some situations, but they also hesitated, avoided certain tasks, tried again after setbacks, and readjusted after ward rotations. The extent of their participation varied with preceptors' judgements, the way feedback was given, patients' responses, and procedural setbacks. Their views of their own clinical ability and of nursing work also changed through these experiences.

**Figure 1:**
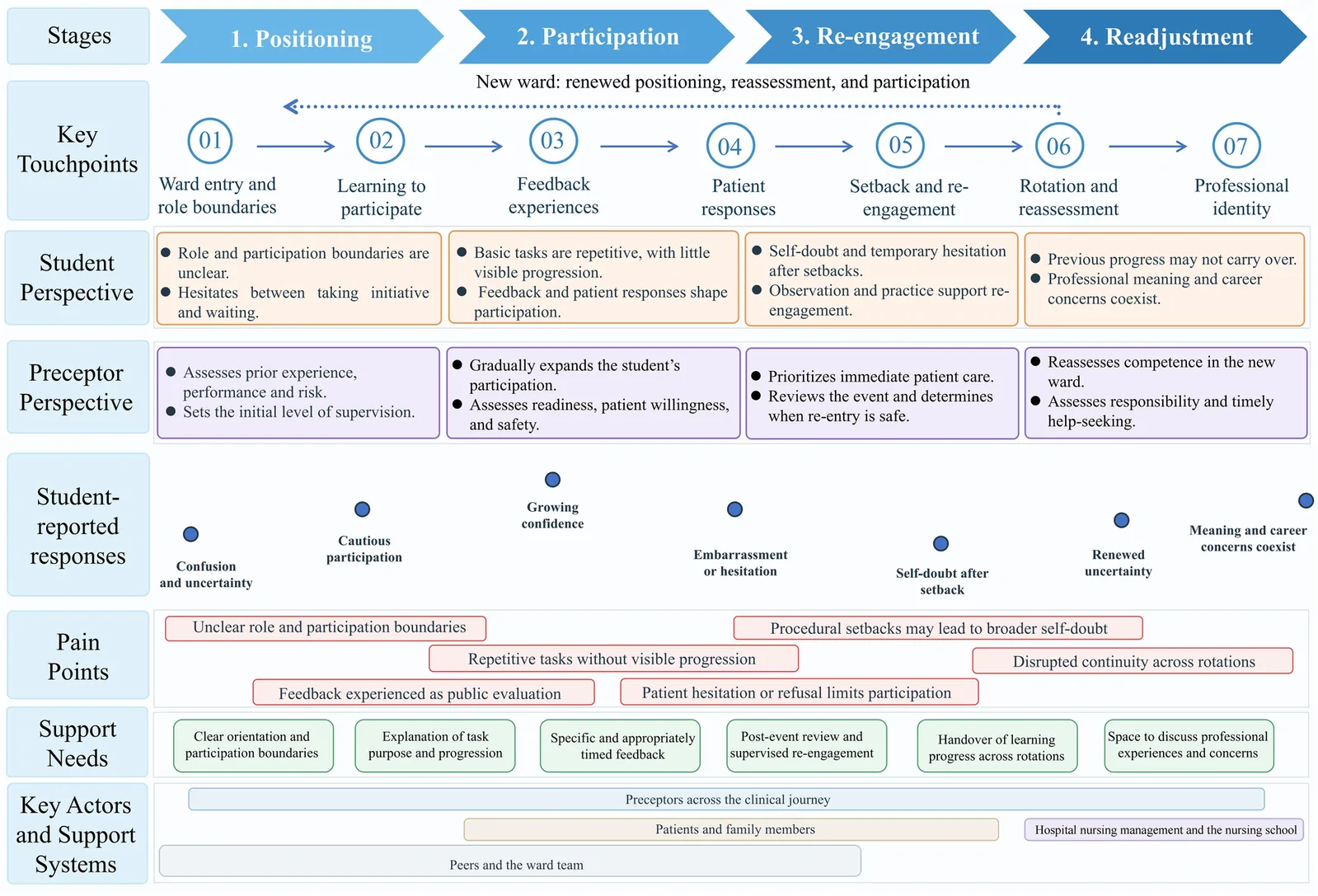
Journey map of clinical adaptation among undergraduate nursing students from student and clinical preceptor perspectives. The vertical positioning of student-reported responses is illustrative and does not indicate quantitatively measured intensity.

### 3.1 Positioning and participation

On entering clinical placement, nursing students needed to clarify their role as learners and the range of tasks in which they could participate, beginning with observation and basic patient care tasks. When taking part in specific tasks, they also needed to apply the knowledge and procedural steps learned at school to clinical situations. Representative quotations are presented in Table 4.

**Table 4:** Subthemes and representative excerpts for theme 1: positioning and participation.

Subtheme	Participant	Representative excerpt
Role Boundaries	Nursing student, S02	“When I first arrived on the ward, I felt a bit lost… The preceptor was busy, and I did not even know where I was supposed to stand. If I went to get something without being asked, I worried she would think I was touching things I should not. If I just followed her around without doing anything, I worried she would think I was not being proactive. Most of the time, it was not that I did not want to do anything. I honestly did not know how far I was allowed to go.”
	Clinical preceptor, T05	“I would never let a new student do something independently straight away. I first need to find out whether she has done it before and whether she knows the steps. Then I decide whether she should watch or do it while I stay beside her. Some students are eager and want to try everything, while others are very cautious. You have to take it step by step.”
Learning to Participate	Nursing student, S11	“At first, I just did whatever the preceptor asked me to do. Most of the time, I was delivering laboratory request forms, collecting medications, making beds, or taking vital signs. At the beginning, I thought these tasks were helping me get familiar with the ward. But after a while, I was still doing the same things, and I started wondering… was I actually learning, or was I just running errands?”
	Clinical preceptor, T03	“I usually let her watch first. Once she is more familiar, she can help with the preparation, and then gradually start doing the procedure while I watch the key steps. Practising at school does not mean she can do it on a real patient straight away. I still have to watch closely, especially during the higher-risk steps.”
	Nursing student, S01	“At school, we practised each procedure as a complete sequence, and it was always clear what came first and what came next. It was different on the ward. A patient might suddenly say they felt unwell, a family member might be asking questions, and the preceptor might need to attend to another patient. I knew the steps, but when several things happened at once… I still didn't know what to deal with first.”

#### 3.1.1 Role boundaries

At the beginning of clinical placement, students often found it difficult to judge their place in the ward and the extent to which they could participate. When clear instructions were lacking, they often hesitated between taking the initiative and waiting to be assigned a task, and adjusted their behaviour based on their preceptors' responses.

Preceptors also judged students' level of participation during clinical teaching. They usually adjusted the level of guidance and supervision according to students’ previous experience, performance in the clinical setting, and the risks associated with the task. Students were more concerned about whether they could actually participate, whereas preceptors placed greater emphasis on students' performance, task risk, and the level of supervision needed when deciding their level of participation.

#### 3.1.2 Learning to participate

Students usually began with basic tasks after entering the ward. When they repeatedly performed similar tasks without clear opportunities for further participation, some began to question whether these tasks were helping them learn. Preceptors, however, viewed observation, assisting with preparation, and performing procedures under supervision as part of gradually increasing students' participation. Students focused more on whether their tasks and opportunities to participate were changing, whereas preceptors focused on whether they were ready to take on more.

Procedural training at school usually followed relatively fixed steps, whereas multiple tasks could occur at the same time in clinical settings. Even when students were familiar with the procedural steps, some still found it difficult to decide which task should be addressed first.

### 3.2 Interaction and adjustment

While participating in patient care, nursing students adjusted their level of involvement through interactions with preceptors, patients, and family members. Feedback, patients' responses, and procedural setbacks could affect whether they continued to participate and whether they were willing to ask questions or seek help. Preceptors also considered patients' preferences and safety requirements when deciding whether students could continue to participate, when guidance was needed, and when they needed to take over and provide feedback. Representative quotations are presented in Table 5.

**Table 5:** Subthemes and representative excerpts for theme 2: interaction and adjustment.

Subtheme	Participant	Representative excerpt
Feedback Experiences	Nursing student, S03	“Once, after handover, my preceptor asked me in front of several other students why a patient was receiving a particular medication. I couldn’t answer… She didn't scold me. She just said, “You should know this.” I was so embarrassed that my face felt hot. After that, whenever I was unsure about something, I wrote it down and looked it up later, or asked another student first. I only went to the preceptor when I still could not work it out.”
	Clinical preceptor, T01	“Some questions have to be asked there and then, especially when the medication has already been prepared or a procedure is about to start. I need to know whether the student understands what the medication is for and what she needs to watch out for. When things are busy, I may speak more directly, but I cannot skip this check just because the student might feel nervous.”
Responses from Patients	Nursing student, S15	“One patient looked at my name badge and said straight away, ‘Let your preceptor do it. I do not feel comfortable with a student.’ My face went red… I knew the patient had the right to choose, but in that moment, I still wondered whether he thought I could not do anything.”
	Nursing student, S04	“Some patients asked how many times I had done the procedure or whether my preceptor would be beside me. As soon as they asked that… I started to hesitate. After that, with patients like this, I usually did not volunteer. I only went over if the preceptor asked me to.”
	Nursing student, S08	“Some patients were very kind. They knew I was a student and still let me have a try. But that made me more nervous… I kept thinking, ‘What if I don't get it right the first time? Will they still let me continue?’ I was so focused on what to do next that I didn’t say much to the patient.”
	Clinical preceptor, T11	“What matters more to me is whether, after entering the room, she notices if the patient is in pain, is worried, or is willing to have her involved. She should not be thinking only about finishing the task I gave her. If the patient does not want her to continue, the first thing she has to do is stop. She cannot keep going just to complete the procedure.”
Setback and Recovery	Nursing student, S09	“Not long after I started clinical practice, I tried to insert an IV cannula for the first time, but I did not succeed. I felt awful because the patient would need another needle… I cried after I left the room. It was not only that I thought I had done badly that time. I started wondering whether I was simply not suited to clinical nursing.”
	Nursing student, S12	“I also had an unsuccessful cannulation. After that, whenever someone needed an IV, I wanted to avoid it… But I knew I could not keep avoiding it, so I watched how my preceptor selected the vein and inserted the needle. I also practised on a training pad during breaks. I was still nervous the next time I tried, but I no longer stepped back straight away as I had before.”
	Clinical preceptor, T08	“When a student makes a mistake for the first time, some of them go pale and just stand there, afraid to move. I do not keep questioning her in front of the patient. I take over and deal with the patient's immediate needs first. After we leave the room, I talk it through with her privately and look at which step went wrong. The patient has to come first. We can talk about teaching afterwards.”

#### 3.2.1 Feedback experiences

Clinical feedback was part of students’ participation in patient care and also affected how they asked questions and sought help afterwards. Students paid attention not only to the content of the feedback, but also to where and how it was given. After being unable to answer a preceptor's question in front of others, some students would first look for information themselves or ask their peers when they were unsure about something, and would ask the preceptor only if they still had questions.

Before medication administration or a nursing procedure, preceptors usually asked questions to check whether students had the necessary knowledge and understood the relevant precautions before deciding whether they could continue to participate. When students talked about these feedback experiences, they more often mentioned how they felt at the time and whether they were still willing to ask questions afterwards, whereas preceptors focused more on students' knowledge and preparation before a procedure and on patient safety.

#### 3.2.2 Responses from patients

Whether patients accepted nursing students’ involvement directly affected students’ opportunities to participate in procedures and patient care. When patients refused their involvement because they were students, some students interpreted the refusal as a lack of trust in their ability. When patients asked about their procedural experience or were cautious about student involvement, some students became more hesitant and were less likely to ask to perform procedures afterwards. Even when patients agreed to their involvement, some students still worried that an unsuccessful attempt might make the patient unwilling to let them continue.

Preceptors considered not only whether students could complete a procedure, but also whether they noticed patients' pain, concerns, or unwillingness to continue. If a patient did not want the student to continue, the preceptor would ask the student to stop the procedure.

#### 3.2.3 Setback and recovery

After an unsuccessful procedure, some students began to doubt their clinical ability and even questioned whether they were suited to clinical work. Others briefly avoided similar procedures, but later observed their preceptors, practised repeatedly, and looked for another opportunity to try. They were still nervous when they tried again, but did not immediately avoid the procedure as they had before.

When a student was unsuccessful or encountered a problem during a procedure, preceptors usually attended to the patient's immediate care needs first. They then reviewed the procedure with the student and discussed the specific problems that had occurred.

### 3.3 Readjustment and identity

Ward rotations brought nursing students into new clinical settings. The level of participation they had already established, and the understanding that previous preceptors had developed of them, did not always carry over to the next ward. In these experiences, students' understanding of nursing was no longer limited to clinical procedures, but also included patient care, communication, and professional responsibility. Representative quotations are presented in Table 6.

**Table 6:** Subthemes and representative excerpts for theme 3: readjustment and identity.

Subtheme	Participant	Representative excerpt
Support Discontinuity	Nursing student, S05	“Every time I moved to a new ward, I had to let the preceptor get to know me all over again. Even with procedures I had already done on the previous ward, I had to start by watching again. I understood that the new preceptor didn’t know what I could do. But sometimes… it felt like the progress I'd made over the past few months didn't carry over. I had to prove myself all over again.”
	Clinical preceptor, T07	“Precepting is not the only thing I have to do that day. I still have patients, medical orders, treatments, and questions from family members to deal with. Sometimes I have more than one student during a shift. When it gets busy, I can only watch the higher-risk procedures closely and let them do the other tasks first. By the time I am free and remember what I wanted to explain, the moment has already passed.”
	Clinical preceptor, T06	“Students in the same group can be very different. Some can start doing it after watching once or twice. Others may know every step by heart but still become nervous when they are in front of a patient. I cannot teach them all at the same pace. I first need to see where each student is getting stuck, whether it is the knowledge, the procedure, or communication with the patient. But when the ward gets busy… it's really hard to watch every student that closely”.
Professional Identity	Nursing student, S04	“Before clinical practice, I thought nursing was mainly about learning how to perform procedures. Once I was actually on the ward, I realized that I also had to observe patients, communicate with family members, and take responsibility for what I did. For a while, I really wondered… is clinical nursing right for me? But sometimes a patient would come and thank you before being discharged, and then I would feel that although the work was tiring, it still meant something.”
	Clinical preceptor, T03	“Later on, I don't look only at whether a student can perform the procedures. Some students are already quite skilled, but when something happens, they still think only about finishing the task in front of them. Others check the patient, report anything unusual, and stop to ask when they're unsure. For me, taking responsibility for the patient matters more than how quickly they finish the task.”

#### 3.3.1 Support discontinuity

After moving to a new ward, students could not always continue at the same level of participation as before. Even if they had already performed certain procedures in a previous ward, they might still need to begin by observing, while the new preceptor assessed their performance before deciding how much they could participate. Some students felt that they had to prove themselves again in the new ward despite the progress they had already made.

In a new ward, preceptors needed time during routine clinical teaching to become familiar with students' performance. When the clinical workload was high, they tended to focus first on higher-risk procedures, which limited the time available for observation, explanation, and feedback. Differences among students in knowledge, procedural skills, and patient communication also made continuous observation and individualised guidance more difficult. Students hoped that their previous learning and performance would carry over to the new ward, whereas new preceptors had limited knowledge of what they had done previously and still needed to decide their level of participation based on what they observed in the current ward.

#### 3.3.2 Professional identity

Clinical care experiences showed students that nursing involved more than completing procedures. It also involved observing patients, communicating with family members, and taking responsibility for the care they provided. Faced with the demands and responsibilities of nursing work, some students questioned whether they were suited to clinical work, while patients' gratitude also helped them see meaning in nursing work.

When preceptors judged whether students were beginning to take on the role of a nurse, they did not focus only on procedural proficiency. They also considered whether students could relate the tasks they were performing to patients' conditions, whether they actively observed patients, and whether they sought help promptly when they noticed abnormalities or were unsure about a situation.

## 4 Discussion

This study examined undergraduate nursing students' clinical adaptation from the perspectives of students and clinical preceptors. Students often judged whether they were adapting to clinical practice by whether they could participate in patient care. Clinical preceptors, in contrast, decided how much students could participate based on their current performance, task risk, patients' preferences, and patient safety. Students' participation did not necessarily increase as the clinical placement progressed, but could expand, become restricted, or be interrupted in different clinical situations. Clinical adaptation involved repeated adjustment over time rather than continuous improvement. Previous studies found that some nursing students were assigned mainly routine or non-nursing tasks during clinical practice and had relatively few opportunities to participate in patient assessment and clinical judgment (10, 39). A gap between students’ expectations of learning opportunities and their clinical role and what they actually experienced may also limit their clinical learning and competence development (19). In this study, students did not question these tasks simply because they were basic. Their concern arose when the same tasks were repeated over time and the link to greater participation remained unclear. Preceptors may view repeated basic tasks as a way to observe whether students follow procedures correctly, perform consistently, and complete the tasks safely under supervision. When the reasons for continuing basic tasks or delaying greater participation were not clear, students could find it difficult to understand how these tasks related to later opportunities to participate. Preceptors also have to balance student participation with patient care responsibilities, limited teaching time, and the pace of work on the ward (40). When clinical work was busy, the reasons behind task allocation were not always explained to students in time, and greater participation was often delayed by conditions on the ward. Based on these findings, when students continued to perform the same type of task or greater participation had to be postponed, preceptors could briefly explain the reason, what they were observing, and what students would need to demonstrate before taking on more. This may help students understand how basic tasks relate to later opportunities to participate.

Students and preceptors described questions asked in the clinical setting differently. Before medication administration or a nursing procedure, preceptors needed to ask questions to check whether students had the necessary knowledge before deciding whether they could participate safely. When these questions were asked in front of their peers, however, students who could not answer might experience them as a public judgement of their ability. For students, the immediate experience was often that they had failed to answer. When students worried about exposing what they did not know, they might ask fewer questions or avoid saying clearly when they were unsure. This could make it harder for preceptors to recognise difficulties in students' knowledge or clinical skills in time (41, 42). Students' willingness to continue seeking feedback may also be shaped by the feedback provider's attitude and the specificity of the feedback (43). Knowledge directly related to patient safety still needs to be checked before medication administration or a procedure. After the procedure, brief and specific feedback could be provided when this did not interfere with patient care. Preceptors could explain what knowledge the student still needed to review or which steps needed adjustment. Such feedback may help students identify specific areas for improvement, rather than leaving them with only a general sense that they did not perform well.

Unlike task allocation and feedback in the clinical setting, patients' acceptance of student involvement cannot be determined simply by adjusting teaching arrangements. Positive interactions with patients can encourage nursing students, strengthen their confidence, and help them recognise the meaning of nursing work (44). Patients are recipients of care, not people whose role is to provide procedural opportunities for clinical teaching. Even when a student is prepared and the preceptor considers supervised participation appropriate, the patient still has the right to refuse (45). Patient refusal should not be attributed simply to poor teaching arrangements, a lack of initiative from the student, or inadequate communication. It is a choice that must be respected in clinical care (46). When patients refused their involvement or asked about their procedural experience, some students began to doubt their own ability and became more hesitant about similar procedures afterwards. When rotations are short or opportunities to perform the same procedure are limited, a single refusal may mean that the student does not have another opportunity to perform it on that ward. Clinical teaching can create learning opportunities, but it cannot guarantee students' participation against a patient's wishes.

After an unsuccessful clinical procedure, nursing students may feel discouraged, doubt themselves, and question their clinical ability. Such experiences do not necessarily translate into learning without opportunities for reflection and further support (47). When the procedure was reviewed and students had another opportunity to try, they were more likely to identify what had gone wrong and make adjustments the next time (48). When a procedure could not be completed, the preceptor usually needed to take over immediately, ensure that the patient's care was completed safely, and review the student's attempt afterwards (49). In a busy clinical setting, however, this follow-up discussion could be delayed. The negative effects of such experiences may continue into later learning, while the support students receive can influence how they understand and respond to what has happened (50). Without further review of the procedure and feedback on the specific problem, students may be left only with a general sense that they “did not do well.” They may then interpret a single setback as evidence that they lack clinical ability more broadly. Even after the problem has been identified, students still need another opportunity to perform a similar procedure under appropriate supervision and adjust what they do based on the previous attempt. When another attempt is not immediately possible, students can still take part in patient assessment, preparation for the procedure, or observation of key steps rather than withdrawing completely from the related care. In this study, recovery after a procedural setback was reflected mainly in whether students were able to re-engage in related clinical practice, rather than in emotional relief alone.

Preceptors preferred students to spend longer periods in the same ward, allowing them sufficient time to become familiar with students' performance (36, 51). This study showed that the difficulty of clinical rotations went beyond becoming familiar with a new environment. Tasks that students had already been allowed to perform and the trust they had built with a preceptor often did not carry over directly to the next ward. The new preceptor still had to decide how much the student could participate based on their performance in the current setting. When assessing a student's ability, preceptors had to consider both the student's performance and their responsibility for patient care (52). Reassessing a student in a new ward did not mean dismissing their previous performance. It was part of the preceptor's responsibility for supervision and patient safety. When information about a student's previous learning was unavailable, preceptors often had to rely on limited observations in the current ward. Students then had to demonstrate abilities they had already developed. Inadequate staffing and limited time for teaching can make it more difficult for clinical nurses to identify students' learning needs (53). This study also found that differences in students' knowledge, clinical skills, and communication with patients made individualised guidance more difficult. Before the new preceptor had formed an initial judgement, students might return to observation, preparation, and basic tasks, while more complex participation was postponed. The aim of improving transitions between rotations is not to remove reassessment in the new ward. Information about the student's previous learning could give the new preceptor a clearer starting point for reassessment. A brief handover could describe the tasks in which the student had already participated, the level of supervision required, and the areas in which further support was needed. Such information would not replace judgements based on the patient's current condition and the clinical situation. As students participated in patient care, received feedback, and experienced procedural setbacks, they also continued to reassess their clinical ability, the responsibilities they were taking on, and whether they were suited to clinical nursing work. Nursing students' professional identity may change over the course of their education, with clinical experiences both strengthening this identity and prompting students to question it (30). Recognition and a sense of achievement during clinical practice may strengthen students' confidence and deepen their understanding of the value of nursing (54). In this study, patients' expressions of gratitude helped some students see meaning in nursing, but did not remove their concerns about the complexity of the work, how demanding it was, and the responsibility involved. The findings suggest that greater attention could be paid to the work-related pressures and career concerns that nursing students face during the middle and later stages of clinical placement. Students could also be given opportunities to express and discuss these concerns.

### 4.1 Strengths and limitations

This study combined students' reflections on their eight-month clinical placement with preceptors' observations in specific wards, showing how the two groups differed in their concerns about task participation, patient safety, and support needs. The journey map was constructed retrospectively from interviews conducted after clinical practice had ended rather than from repeated data collection at each stage. Students' later experiences may therefore have influenced how they recalled and interpreted earlier events and emotions. Participants’ responses may also have been influenced by social desirability bias when students described their clinical placement and preceptors described their precepting experiences. As participation was voluntary, participants who contacted the research team may have differed from non-participants in their willingness to share clinical experiences. Students and preceptors were not individually matched, and preceptors usually worked with students during only one clinical rotation. The study could therefore compare shared concerns and differences across the two datasets, but could not determine whether a particular student and preceptor understood the same event in similar ways. The study was conducted in one tertiary Grade A general teaching hospital in Anhui Province, China, and all student participants were from the same cohort. Clinical education settings may differ in ward rotation arrangements, approaches to clinical preceptorship, and patients' acceptance of nursing students' involvement in care. These differences may affect students' opportunities to participate and the continuity of support. They should be considered when interpreting the findings in other clinical education settings. Future studies could conduct repeated interviews at different stages of clinical practice and include direct observation or matched student–preceptor data to provide a more continuous account of changes in students' clinical adaptation.

## 5 Conclusions

This study found that undergraduate nursing students' clinical adaptation did not follow a steady course during clinical placement, but involved repeated adjustment as they participated in patient care, received feedback, experienced procedural setbacks, and moved between wards. Opportunities to participate could be restricted or interrupted in different clinical situations and later re-established. Clinical education should provide specific feedback and opportunities for students to re-engage, and improve the continuity of learning information across ward rotations while maintaining patient safety.

## Data Availability

The original contributions presented in the study are included in the article/Supplementary Material, further inquiries can be directed to the corresponding author/s.
