# Supplementary material for: Undergraduate nursing students' clinical adaptation: a qualitative journey mapping study from student and clinical preceptor perspectives
Source: Front Med (Lausanne). 2026 Oct 9;13:1966449. doi: 10.3389/fmed.2026.1966449 (PMC13690658; doi:10.3389/fmed.2026.1966449)
Supplement: Supplementary file 1 [file Datasheet1.pdf]

Supplementary File 1: Consolidated criteria for reporting qualitative studies (COREQ): 32-item checklist

Adapted from: Tong A, Sainsbury P, Craig J. Consolidated criteria for reporting qualitative research (COREQ): a 32-item checklist for interviews and focus groups. International journal for quality in health care. 2007; 19(6):349-57.

Item	Guide questions/description	Reported on Page
**Domain 1: Research team and reflexivity**		
Personal Characteristics		
1. Inter viewer/facilitator	Which author/s conducted the interview or focus group?	Pg 4 (Methods)
2. Credentials	What were the researcher's credentials? E.g. PhD, MD	N/A
3. Occupation	What was their occupation at the time of the study?	Pg 4 (Methods)
4. Gender	Was the researcher male or female?	Pg 4 (Methods)
5. Experience and training	What experience or training did the researcher have?	Pg 4 (Methods)
Relationship with participants		
6. Relationship established	Was a relationship established prior to study commencement?	Pg 4 (Methods)
7. Participant knowledge of the interviewer	What did the participants know about the researcher? e.g. personal goals, reasons for doing the research	Pg 5 (Methods)
8. Interviewer characteristics	What characteristics were reported about the interviewer/facilitator? e.g. Bias, assumptions, reasons and interests in the research topic	Pg 4–5 (Methods)
**Domain 2: study design**		
Theoretical framework		
9. Methodological orientation and Theory	What methodological orientation was stated to underpin the study? e.g. grounded theory, discourse analysis, ethnography, phenomenology, content analysis	Pg 3 and 5 (Methods)
Participant selection		
10. Sampling	How were participants selected? e.g. purposive, convenience, consecutive, snowball	Pg 4 (Methods)

11. Method of approach	How were participants approached? e.g. face-to-face, telephone, mail, email	Pg 4 (Methods)
12. Sample size	How many participants were in the study?	Pg 4 (Methods) & Pg 16 (Table 1)
13. Non-participation	How many people refused to participate or dropped out? Reasons?	Pg 4 (Methods)
Setting		
14. Setting of data collection	Where was the data collected? e.g. home, clinic, workplace	Pg 5 (Methods)
15. Presence of non-participants	Was anyone else present besides the participants and researchers?	Pg 5 (Methods)
16. Description of sample	What are the important characteristics of the sample? e.g. demographic data, date	Pg 3 (Methods) & Pg 16 (Table 1)
Data collection		
17. Interview guide	Were questions, prompts, guides provided by the authors? Was it pilot tested?	Pg 4 (Methods) & Pg 17 (Table 2)
18. Repeat interviews	Were repeat interviews carried out? If yes, how many?	Pg 4 (Methods)
19. Audio/visual recording	Did the research use audio or visual recording to collect the data?	Pg 5 (Methods)
20. Field notes	Were field notes made during and/or after the interview or focus group?	Pg 5 (Methods)
21. Duration	What was the duration of the interviews or focus group?	Pg 5 (Methods)
22. Data saturation	Was data saturation discussed?	Pg 4 (Methods)
23. Transcripts returned	Were transcripts returned to participants for comment and/or correction?	Pg 5 (Methods)
**Domain 3: analysis and findings**		
Data analysis		
24. Number of data coders	How many data coders coded the data?	Pg 5 (Methods)
25. Description of the coding tree	Did authors provide a description of the coding tree?	N/A
26. Derivation of themes	Were themes identified in advance or derived from the data?	Pg 5 (Methods)
27. Software	What software, if applicable, was used to manage the data?	Pg 5 (Methods)
28. Participant checking	Did participants provide feedback on the findings?	Pg 6 (Methods)
Reporting		
29. Quotations presented	Were participant quotations presented to illustrate the themes/findings? Was each quotation identified? e.g. participant	Pgs 18–20 (Tables 4–6)

	number	
30. Data and findings consistent	Was there consistency between the data presented and the findings?	Pgs 6–9 (Results) & Pgs 18–20 (Tables 4–6)
31. Clarity of major themes	Were major themes clearly presented in the findings?	Pg 6–9 (Results) & Pg 17 (Table 3)
32. Clarity of minor themes	Is there a description of diverse cases or discussion of minor themes?	Pgs 7–9 (Results) & Pgs 18–20 (Tables 4–6)
